# Patellar Reconstruction During Total Knee Arthroplasty for Previous Patellectomy

**DOI:** 10.1016/j.artd.2025.101944

**Published:** 2026-05-01

**Authors:** Chun Man Lawrence Lau, Ka Chun Thomas Leung, Michelle Hilda Luk, Amy Cheung, Kwong Yuen Chiu, Henry Fu, Ping Keung Chan

**Affiliations:** a Department of Orthopaedics and Traumatology, School of Clinical Medicine, The University of Hong Kong, Hong Kong SAR, China; b Department of Orthopaedics and Traumatology, Queen Mary Hospital, Hong Kong SAR, China

**Keywords:** Neo-patella, Patellectomy, Patellar reconstruction, Malalignment

## Abstract

Patellectomy, an uncommon procedure in the current era, poses a challenge in total knee arthroplasty (TKA), creating anteroposterior instability, anterior knee pain, and loss of mechanical advantage. The TKA is even more complicated when the patellectomy is performed secondary to lower limb malalignment with patellofemoral pain and instability. Here, we describe a case of patellectomy and malalignment, and the surgical technique for patellar reconstruction with concomitant TKA to address this complex scenario.

## Introduction

Patellectomy is an uncommon procedure in the current era, as it was found in the 1970s that it would lead to accelerated tibiofemoral degeneration owing to excessive force required to achieve full knee extension when the patella is removed [1,2]. Before these problems became apparent, patellectomy was used as a modality to manage severe anterior knee pain or patellofemoral joint dysfunction [3]. “Miserable malalignment”, a term popularized by Stanley James in 1979, describes a combination of excessive femoral anteversion, patella alta, and excessive outward tibial rotation leading to anterior knee pain [4]. With an increasing understanding of this valgus and rotational malalignment over the years, surgical intervention now focuses on osteotomies of the femur and tibia to correct the malalignment, instead of simply removing the patella [4].

However, patellectomy was used for anterior knee pain secondary to malalignment at a time before the pathoanatomy of the valgus and rotational malalignment was well articulated, and now these patients with patellectomy have grown older with progressive degeneration occurring at their knees. Total knee replacements (TKRs) for osteoarthritis in these knees are challenging as the lack of patella leads to anteroposterior instability, anterior knee pain, and loss of mechanical advantage of the knee extensor mechanism [5,6]. Anteroposterior instability is a commonly quoted condition following patellectomy [7], and it has also been reported in cases with TKR that subsequently underwent patellectomy [7]. During TKR, malrotation and malalignment would be aimed to be corrected; however, a residual patellar tracking problem is still possible due to chronically misaligned muscle vectors, for example, wasting of the vastus medialis, and or soft tissue contracture, which may not be fully corrected after TKR and its soft tissue procedure [8]. Reconstruction of the patella is difficult even after correcting malalignment of the tibia and femur with TKR, as chronically misaligned muscle force vector hampers the osteointegration of the new patella, “neopatella”. Various patellar reconstruction methods exist with the use of different graft choices as the neopatella, each with its pros and cons. [6,[9], [10], [11]]

Here, we present a case in which we carefully planned the patellar reconstruction with modifications from previous descriptions during TKR to tackle an osteoarthritic knee with patellectomy and malalignment. To the best of our knowledge, the technique presented has not been described before, and the case scenario is rare.

## Case history

A 74-year-old female was referred to our team for progressive right knee pain with a reduction in knee range. Informed consent (with electronic system documentation) was obtained from the patient for discussing her clinical information. History taking revealed that she had undergone a total patellectomy between her 20s and 30s for debilitating anterior knee pain, and she was not certain whether any patellar dislocation occurred at that time, but she confirmed that she had never suffered from a patellar fracture. She has recovered well after patellectomy except for mild extension weakness, but she gradually developed right knee pain with progressive extension weakness over the past 2 years and became trolley/frame-dependent on walking and swung her knee during walking to compensate for the weakness. A hinged knee brace locked in extension during walking was given to prevent falls. She had severe knee pain and could only tolerate walking on level ground for 10 minutes.

**Figure 1:**
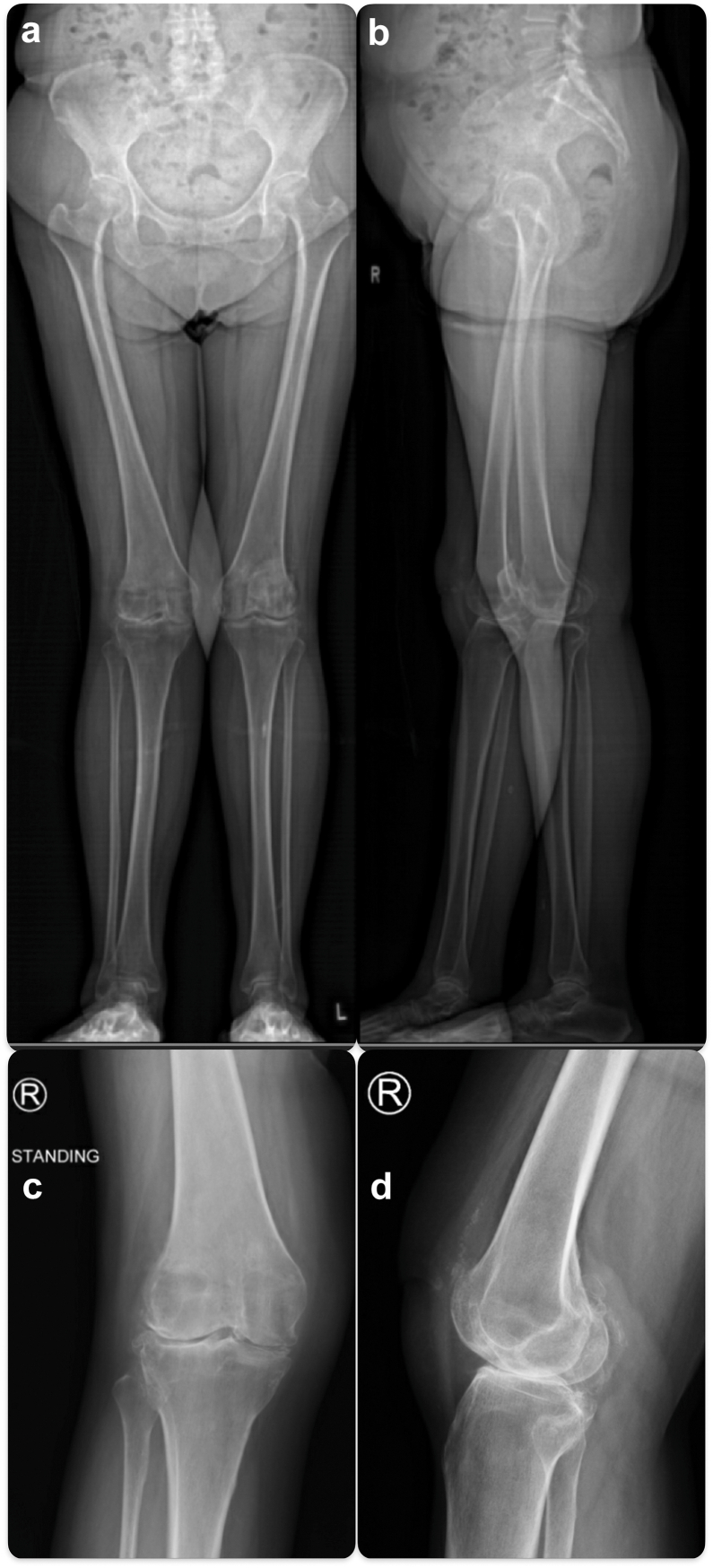
(a) EOS anteroposterior view. (b) EOS lateral view. (c) Knee anteroposterior view. (d) Knee lateral view. There was an absence of patella and valgus alignment in both lower limbs.

**Figure 2:**
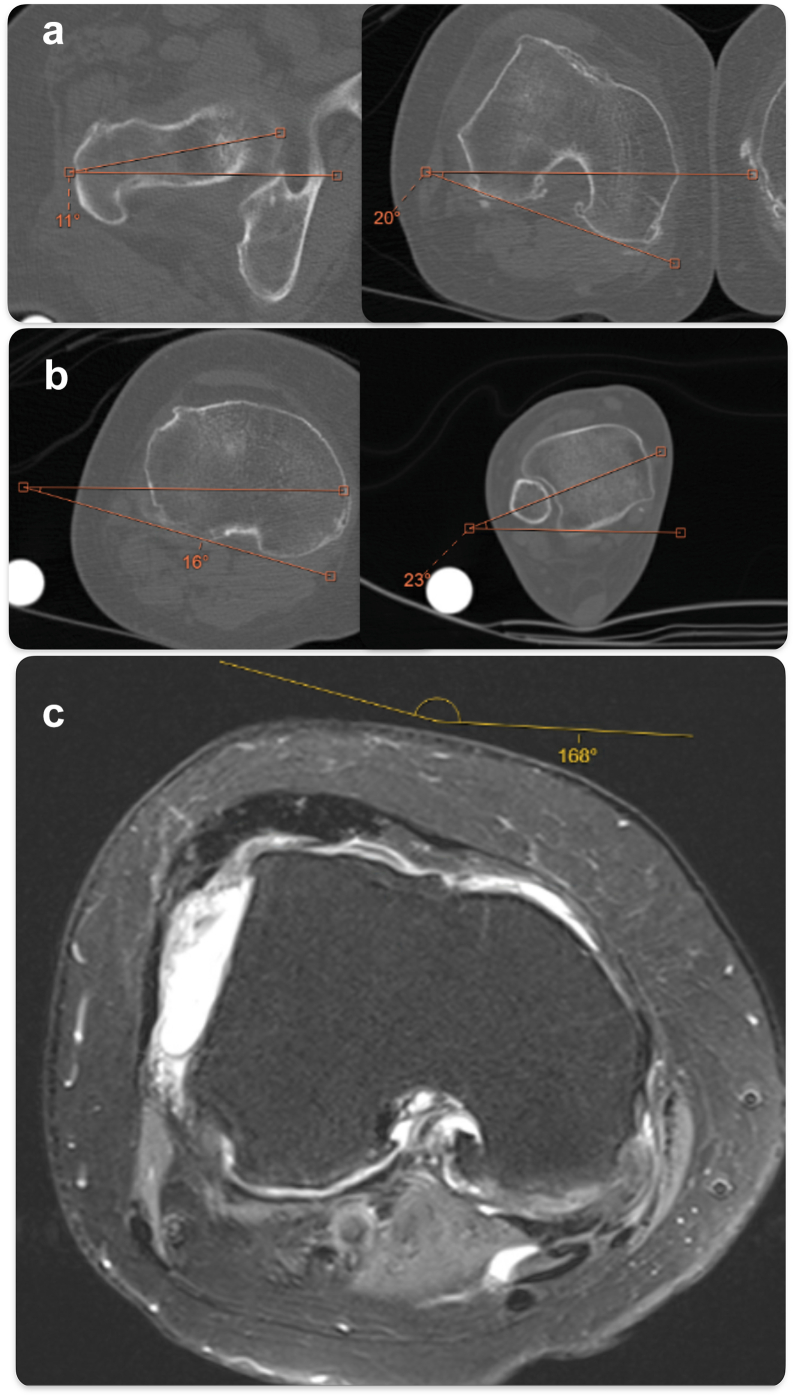
(a) CT femur anteversion, (b) CT tibial torsion, (c) MRI axial cut of the knee. Excessive femoral anteversion and tibial torsion were noted, with trochlear dysplasia on MRI and lateralization of the extensor mechanism. CT, computed tomography.

Physical examination showed that she had bilateral valgus knees with inward-pointing patella on the left side and a transverse knee scar on the right side. She had 70° extension lag (active range 70-110°), and the passive knee range was 0-110° with poor extensor strength (Medical Research Council Scale 2/5). Tenderness was localized to the tibiofemoral joint only. Increased clinical thigh–foot angle was observed. We used EOS imaging to obtain a simultaneous biplanar weight-bearing view of the whole lower limb, showing the right mechanical tibiofemoral angle of 13° valgus [12] (Fig. 1). Magnetic resonance imaging (MRI) showed trochlea dysplasia of sulcus angle 168° of type C in Dejour classification and severe in Oswestry-Bristol classification. [13,14] Computed tomography showed femoral anteversion of 31° and tibial torsion of 39° (Fig. 2). These findings were compatible with valgus and rotational malalignment as previously reported [4]. In addition, we performed the MRI as there was no formal documentation about the total patellectomy done between her 20s and 30s, and the MRI allowed us to assess the integrity of the patella tendon, quadricep tendon, and quadricep muscle, which were all important in the outcome of a neopatella reconstruction and whether we could longitudinally split the structures as described subsequently. The Knee Society Knee Score was 47, and the Knee Society Function Score was 0 before the operation.

**Figure 3:**
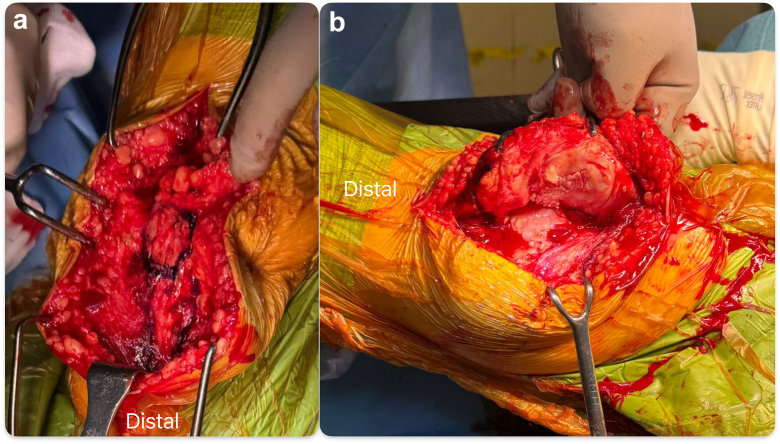
(a) Tissue hardening noted before arthrotomy. (b) The back of the hardening was identified as scar tissue from the previous patellectomy.

**Figure 4:**
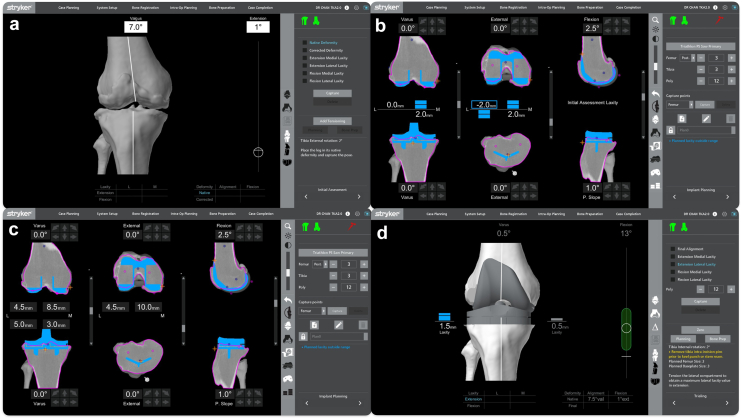
(a) Image-based robotic TKR workflow screen capture. (a) Assessment of alignment showed valgus 7° after osteophyte removal. (b) Balancing components are performed based on the digital tensioner. (c) Balancing components were performed with a bone cut shown, which also reflected the size of the autograft. (d) After component insertion, medial and lateral laxity were measured with a digital tensioner.

**Figure 5:**
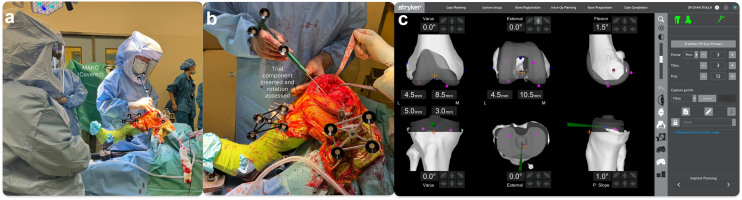
(a) Registration and execution of Image-based robotic TKR. (b) Bone cut was performed with Image-based robotic TKR, and tibial tray trial component rotation was assessed with a probe. (c) Rotation assessment as shown on the workflow screen.

TKR with patellar reconstruction was offered and performed. A midline incision perpendicular to the previous transverse scar was used, and the site of hardening corresponding to the previous patellectomy site was identified. A medial parapatellar approach, medial to the hardening, was used, and a circular scar at the back of the hardening was noted. Possible patella alta before patellectomy was appreciated from the scar position (Fig. 3). We aimed for a neutral mechanical alignment during the initial planning using an image-based robotic-arm-assisted TKR (Stryker) workflow. During the planning, we also sized the resected distal femur condyle for use as a neopatella autograft and externally rotated the femoral and tibial components to minimize the effect of native rotational malalignment (Figs. 4 and 5). Due to valgus malalignment with chronic stretching and elongation of the medial collateral ligament, we noted there was medial laxity (∼2.5 mm) and lateral tightness (∼0 mm) in flexion and extension gap from the digital tensioner initially. A bony cut was then performed with the robotic saw. Trial components with a posterior-stabilized insert were inserted and tested. We further released lateral tightness following Whiteside et al. technique, correcting the lateral side to ∼1.5 mm laxity in flexion and extension, while the medial side with remaining 2.5 mm laxity. Trialing with a total stabilizer insert was able to correct medial and lateral laxity to between 0-1 mm on both medial and lateral sides.

**Figure 6:**
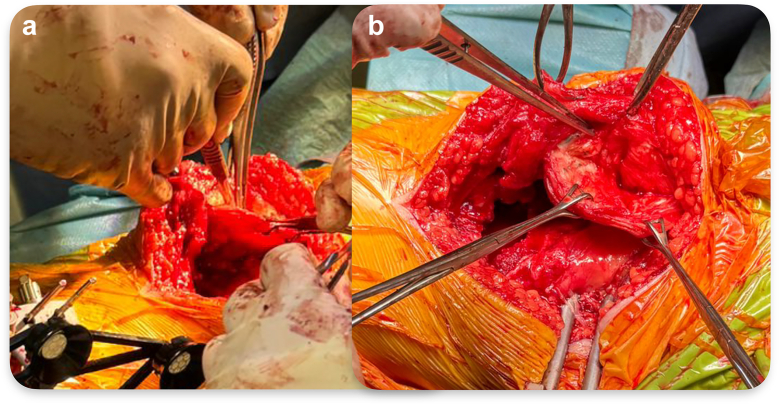
(a) Dissection of scar tissue to create a pocket. (b) Pocket created.

**Figure 7:**
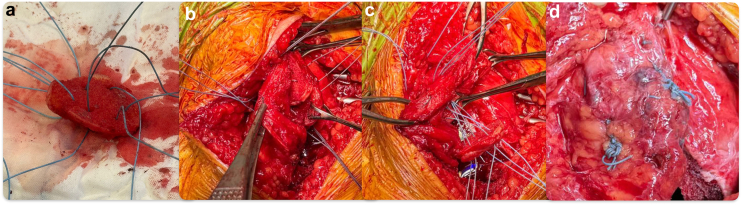
(a) Autograft with suture passed. (b) Autograft placed in the pocket. (c) Suture passed anteriorly and posteriorly through the pocket. (d) Interlocking suture tied to secure autograft.

**Figure 8:**
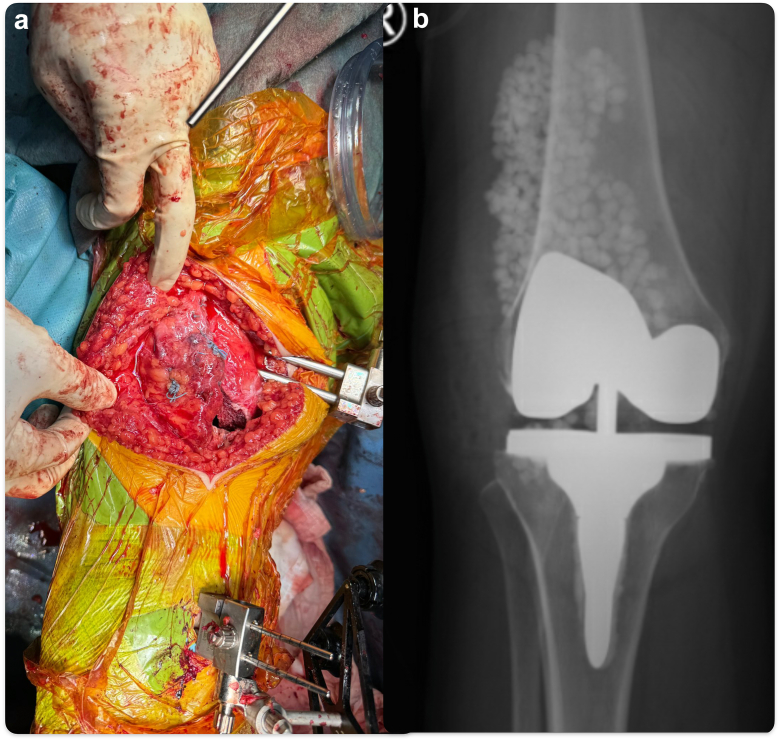
(a) Neopatella final appearance (b) Calcium sulfate beads inserted.

The patellar reconstruction was performed by longitudinally splitting the quadriceps, suprapatellar bursa, and the residual scar tissue of the patellectomy from medial to lateral along the coronal plane using the medial parapatellar approach incision, to preserve an intact thin layer of scar tissue envelope in 1 piece facing the femoral component. This allowed the whole layer of tissue facing the joint cavity, from the patellar tendon to scar tissue to quadricep tissue, to be continuous and left untouched. A pocket was gradually developed with the coronal dissection of the scar tissue and suprapatellar bursa/quadricep tendon (Fig. 6). The resected distal femur condyle was trimmed into a patella shape as the neopatella, with multiple sutures passing through its periphery. This neopatella was then placed at the central lower end of the scar tissue pocket with TiCron sutures (Covidien, Dublin, Ireland) passing through both the anterior and posterior sides of the scar tissue pocket (Fig. 7). Bioabsorbable calcium sulfate beads (Stimulan, Biocomposites Ltd, Keele, United Kingdom) mixed with vancomycin were placed inside (smaller portion) and outside of the scar tissue pocket [15]. The TiCron sutures were tied in an interlocking fashion to secure the neopatella position and close the space in the pocket (Fig. 8). Patellar tracking was confirmed satisfactory using the no thumb technique by flexing the knee from 0° to 90° without any lateral force applied by the surgeon’s thumb to prevent the patella from subluxation laterally and the patellar tracking assessment method described by Batailler et. al. [16] Using the Batailler et. al. method, the neopatella was reduced in front of the femur and the central position of the neopatella were repeatedly captured using the probe of the image-based robotic-assisted TKR system at every 10° of flexion between the full extension and 90° knee flexion to visualize the patellar tracking [16]. The tracking was adjusted by changing the position of the neopatella through closing the lateral space of the scar tissue pocket and adjusting the external rotation of the tibial baseplate. We used the probe of the image-based robotic-assisted TKR system to visualize the axis of the tibial tray to confirm its position relative to the Akagi line (the center of the posterior cruciate ligament to the medial border of the tibial tuberosity) [17] and marked the central axis of tray during each succession change of its position with diathermy to ensure improvement in external rotation of tibial tray. [18]

Final TKR components (Triathlon PS femur, tibial tray with short, cemented stem, and total stabilizer insert, (Stryker)) were inserted. Anteroposterior stability was confirmed clinically and from the virtual model in the robotic system. Arthrotomy was closed together with vastus medialis obliquus (VMO) advancement to the mid-to-distal aspect of neopatella as described by Insall et al. in a pants-over-vest manner. [19] Knee range 0-125° was achieved intraoperatively. The wound was covered with a negative-pressure dressing. [20,21]

**Figure 9:**
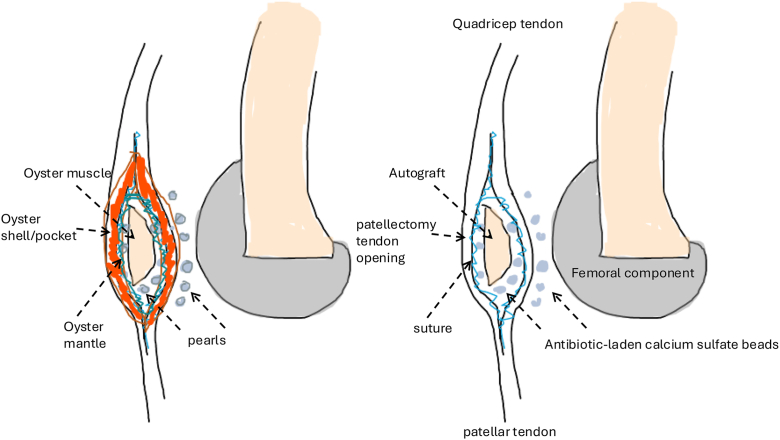
Schematic figure of the neopatella configuration.

The patient was allowed immediate full weight-bearing and full knee range mobilization as tolerated postoperatively. On follow-up at 2 years, she could walk unaided for more than 1 hour and achieve an active knee range of 0-125° with full extension power (Videos 1 and 2). The Knee Society Knee Score was 100, and the Knee Society Function Score was 70. The patient has no anterior knee pain during level ground walking, stairs ascending and descending, and kneeling. Radiograph showed TKR and neopatella in proper positioning without resorption or any other complications (Fig. 9).

## Discussion

Our neopatella technique featured several points that were different from previous reports. [6,10,[22], [23], [24]]. While there were other previous publications mentioning the utilization of a soft tissue pocket, however, the format and style of the creation were different from ours. For example, in the classical publication by Abdel et al. [25], the method involved placing a bone graft on the ventral side of the quadricep tendon and using another soft tissue graft, like fascia lata, to cover up the bone graft while suturing the periphery of the fascia lata to keep the bone graft in place. The soft tissue pocket we used in this case was developed differently. We aimed to completely preserve articular side tissue, so we developed a pocket from the medial side longitudinally, which is different from previous reports, which commonly suture an allograft, autograft, or patellar prosthesis directly onto the articular side of the patellectomy tendon, and we partially denuded cartilage at the periphery of the autograft. This has the advantage of allowing the autograft to achieve healing from both the anterior and posterior sides. It avoided potential displacement or formation of a loose body even if the autograft resorbed and failed integration, as it was enclosed in the closed pocket of the patellectomy tendon. We thought the major advantage was that this configuration would be significantly less likely to lead to the formation of a loose body compared to other configurations (eg, the fascia lata and the bone grafts could be dislodged if the sutures break prematurely when the suture and fascia lata rubbed against the trochlear groove. In our configuration, the ventral side was the native scar tissue, which was stronger as it was intact longitudinally. So during flexion–extension, it was not just the sutures acting against the displacement force, but the native scar and reinforcement sutures. And we used an intact bone chip as a neopatella, which was sized with a robotic-assisted TKR system. In our case, we corrected a valgus, rotationally misaligned knee into neutral mechanical alignment with a rotational profile optimized and prepared an adequately sized autograft as a neopatella. Image-based robotic TKR would allow planning the size of the neopatella autograft before it is cut. Nevertheless, these sizes may also be achievable by experts by using calipers repeatedly.

In a previous publication by A. D. Hanssen [11], he used cancellous bone graft to replenish patellar bone defect during revision knee arthroplasty, as it is difficult to have a good quality bone piece during revision. In our situation, we were performing a primary TKR, and the availability of a strong intact bone piece was of less concern. We selected the distal femur bone piece, which consisted of strong and hard subchondral bone with high bone mineral density over that region. This single piece of bone would thus have a higher chance to maintain its structural integrity with less resorption compared to a cancellous bone graft alone. However, the limitation would be the limited “volume/size” of the eventual neopatella, and a potential future method of combining cancellous bone graft with an intact single bone piece may potentially have the advantage of both methods, like increasing the size of the neopatella. We also deliberately positioned the neopatella in the mid-layer of the quadricep/patella tendon since the patella is developed from the mid-substance of the quadricep/patella tendon according to an embryology study. [26] Therefore, the neopatella would be in a more natural anatomical layer and avoid overstuffing the patellofemoral joint, reducing the flexion range. The final appearance of our patellar reconstruction resembled an “*oyster* (*oyster mantle:* suture; *oyster muscle:* autograft) in the pocket (*oyster shell*/pocket: patellectomy tendon opening)”, with multiple calcium sulfate beads, like *oyster pearls*, providing vancomycin to reduce infection risk and calcium to facilitate autograft integration (Figs. 8 and 9). [15,27,28]

The preoperative MRI was useful as it allowed us to assess the thicknesses and integrities of the patella tendon, the patellectomy scar tissue, the quadriceps tendon, and the quadriceps muscle. These allowed us to gauge whether longitudinally splitting the structures as described in the above technique would be safe without causing an excessively thin patella/scar/quadricep tendon that would easily rupture after splitting. In other words, if the preoperative MRI found the patella/scar/quadricep tendon was very thin, this technique was contraindicated.

In this technique, we continued to use the patient’s pre-existing articulation of scar tissue onto the trochlea as the final articulating surface, except that we changed the trochlear side to the femoral component. This technique has a potential risk of pain with the soft tissue articulation. Before the operation, we observed the site of tenderness localized to the tibiofemoral joint instead of the anterior part of the knee, and therefore, we deduced that the scar tissue articulating with the trochlea did not cause pain. We hypothesized that these were mainly scar tissue from previous surgery without nerve endings, and therefore did not cause pain. Besides, if soft tissue articulation would lead to pain, then the patient would have developed pain soon after patellectomy, but not until the recent deterioration of knee osteoarthritis. Therefore, based on these logics, we proceeded to use the scar tissue as part of the neopatella creation. Two years after the operation, she did not have any anterior knee pain, and she was able to fully flex her knee as shown in the video and kneel as tolerated. Besides, given the chronic elongation of medial collateral ligament and residual imbalance of the medial-lateral laxity after lateral release, we used a varus–valgus constraint insert to ensure mediolateral stability.

**Figure 10:**
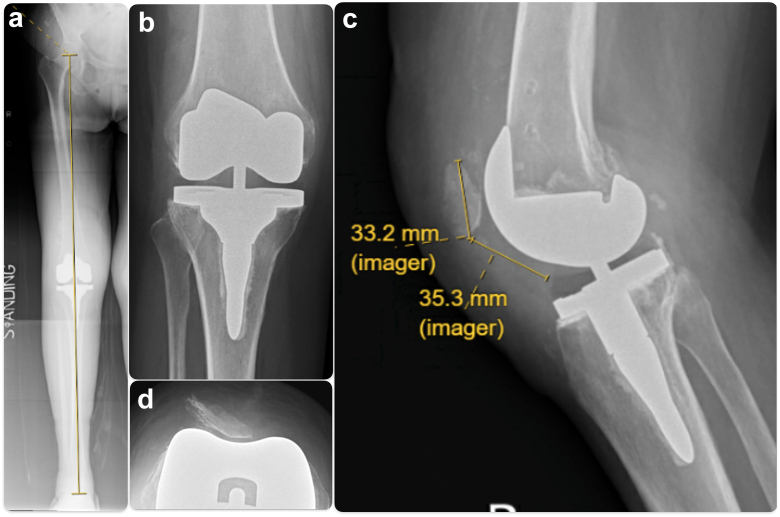
(a) Neutral mechanical alignment achieved. (b) TKR anteroposterior view (c) TKR lateral view (d) TKR skyline view.

Although we present a successful surgical technique in managing osteoarthritic knee with previous patellectomy and malalignment, we have to highlight that patellofemoral joint pain and dysfunction are quite common among adolescents and young adults, and patellectomy should not be the preferred option for the management of anterior knee pain nowadays [1,4,19,29,30]. Joint replacement should only be used as a salvage option for osteoarthritic joints, but not as an option to correct malalignment alone due to potential complications in the long run [[31], [32], [33], [34], [35]]. The treatment options for this patient included TKR without patellar reconstruction. However, in previous cohorts of TKR with subsequent patellectomy performed, 25% of them had instability and 50% had extension lag, so this option was not chosen in our case, as she also suffered from reduced extensor strength after patellectomy [7]. Even though we have corrected coronal and rotational malalignment, and centralized autograft position with respect to the trochlear groove of TKR, together with Insall VMO advancement in our operation, there was still mild patella tilt in the skyline view radiograph in the latest follow-up (Fig. 10). This did not create clinical symptoms in our patient, but this radiological appearance informs us that the patient’s VMO was weak secondary to long-term lack of use after patellectomy (or atrophic as she was with malalignment). Therefore, a lateral parapatellar approach, allowing better release of lateral structure, may be more advantageous in this scenario if VMO advancement is deemed to be less useful [36]. However, the current setting of image-based robotic-arm–assisted TKR does not allow a lateral parapatellar approach to be used, and therefore we could not use a lateral parapatellar approach. Instead, we performed VMO advancement to improve the force vector of the neopatella.

## Summary

Performing TKR in the knee with a previous patellectomy is challenging. It is even more complicated when the patellectomy is performed secondary to lower limb malalignment with patellofemoral pain and instability. We combined robotic-arm TKR with the novel “oyster in pocket” neopatella technique to tackle this challenging scenario in TKR.

## Informed patient consent

This study has obtained patient informed consent and institutional review board ethical approval (with ethical approval number: HKWC-2025-506 UW 25-485).

## CRediT authorship contribution statement

**Chun Man Lawrence Lau:** Writing – review & editing, Writing – original draft, Methodology, Investigation, Conceptualization. **Ka Chun Thomas Leung:** Project administration. **Michelle Hilda Luk:** Project administration. **Amy Cheung:** Project administration. **Kwong Yuen Chiu:** Supervision, Resources, Funding acquisition. **Henry Fu:** Supervision, Resources, Project administration. **Ping Keung Chan:** Writing – review & editing, Supervision, Resources, Project administration, Funding acquisition, Data curation, Conceptualization.

## Conflicts of interest

Professor Kwong Yuen Chiu is a paid consultant for Depuy, Johnson and Johnson Smith and Nephew Stryker Mako Zimmer Biomet Microport.

Professor Ping Keung Chan is in the American Association of Hip and Knee Surgeons International Committee.

The other authors declare no potential conflicts of interest.

For full disclosure statements refer to https://doi.org/10.1016/j.artd.2025.101944.

## Appendix A Supplementary data

Video 1 Patient is walking unaided, and the transverse and vertical scar on the right knee can be seen on closer inspection.

Video 2 Patient can achieve active full extension and flexion.

Conflict of Interest Statement for Fu

Conflict of Interest Statement for Chan

Conflict of Interest Statement for Cheung

Conflict of Interest Statement for Chiu

Conflict of Interest Statement for Lau

Conflict of Interest Statement for Leung

Conflict of Interest Statement for Luk
